# Analyses of the Erosive Effect of Dietary Substances and Medications on Deciduous Teeth

**DOI:** 10.1371/journal.pone.0143957

**Published:** 2015-12-23

**Authors:** Adrian Lussi, Thiago Saads Carvalho

**Affiliations:** Department of Preventive, Restorative and Pediatric Dentistry, University of Bern, Bern, Switzerland; New York Institute of Technology College of Osteopathic Medicine, UNITED STATES

## Abstract

This study aimed at analysing the erosive potential of 30 substances (drinks, candies, and medicaments) on deciduous enamel, and analyse the associated chemical factors with enamel dissolution. We analysed the initial pH, titratable acidity (TA) to pH 5.5, calcium (Ca), inorganic phosphate (P_i_), and fluoride (F) concentration, and degree of saturation ((p*K* -p*I*)_HAP_, (p*K* -p*I*)_FAP_, and (p*K*−p*I*)_CaF2_) of all substances. Then, we randomly distributed 300 specimens of human deciduous enamel into 30 groups (n = 10 for each of the substances tested. We also prepared 20 specimens of permanent enamel for the sake of comparison between the two types of teeth, and we tested them in mineral water and Coca-Cola^®^. In all specimens, we measured surface hardness (VHN: Vickers hardness numbers) and surface reflection intensity (SRI) at baseline (SH_baseline_ and SRI_baseline_), after a total of 2 min (SH_2min_) and after 4 min (SH_4min_ and SRI_4min_) erosive challenges (60 ml of substance for 6 enamel samples; 30°C, under constant agitation at 95 rpm). There was no significant difference in SH_baseline_ between deciduous and permanent enamel. Comparing both teeth, we observed that after the first erosive challenge with Coca-Cola^®^, a significantly greater hardness loss was seen in deciduous (−90.2±11.3 VHN) than in permanent enamel (−44.3±12.2 VHN; p = 0.007), but no differences between the two types of teeth were observed after two challenges (SH_4min_). After both erosive challenges, all substances except for mineral water caused a significant loss in relative surface reflectivity intensity, and most substances caused a significant loss in surface hardness. Multiple regression analyses showed that pH, TA and Ca concentration play a significant role in initial erosion of deciduous enamel. We conclude that drinks, foodstuffs and medications commonly consumed by children can cause erosion of deciduous teeth and erosion is mainly associated with pH, titratable acidity and calcium concentration in the solution.

## Introduction

Dental erosion is the acid dissolution of dental hard tissues caused by multiple factors. One of these factors are acidic substances in the diet (nutrition-related factors) [1]. Erosion can occur in both deciduous and permanent teeth [2–5]. It starts with a softening of the tooth surface (enamel) and progresses to extensive loss of tooth substance when contact with the acids continues [6–8]. Various dietary substances and medicaments have been associated with dental erosion [9–15], and many studies have investigated which chemical factors are most significantly associated with enamel dissolution [16–23]. However, many studies have focused on permanent teeth, and more detailed investigations should be carried out to find out the effect of different dietary substances on deciduous enamel, and which chemical factors will play a role on erosive demineralization of these teeth.

Deciduous enamel is histologically different to permanent enamel. Basically, prism arrangements in deciduous and permanent enamel are similar [24], but the prisms in deciduous enamel are smaller, with more complete boundaries, and are more widely spread than those in permanent enamel [25]. Also, the prisms in deciduous enamel are more gently curved, and have slightly less pronounced Hunter-Schreger bands [25]. Deciduous enamel is considerably less mineralized [26], has greater total carbonate content [27], and a higher organic content [28] than permanent enamel. These histological differences could also lead to different erosion patterns in deciduous and permanent enamel, so it is important to fully investigate the effect of different dietary substances on deciduous enamel.

Moreover, in a study by Ganss et al. [29], children who initially presented with erosive lesions in deciduous teeth had a significantly greater risk (3.9-fold) of having erosive lesions in their permanent teeth. Similar results were also reported by Harding et al. [30], who showed that 5-year-old children who present with severe erosive tooth wear in deciduous teeth are 5 times more likely to present erosive tooth wear in permanent teeth at the age of 12 years. It is, thus, suggested that tooth wear in deciduous teeth ought to be regarded as a predictive factor for wear in permanent teeth, and health professionals should be fully aware of the erosive effect of different dietary substances on deciduous enamel in order to be able to give children and parents the best oral health recommendations. Consequently, the aim of this study was to analyse the potential of different substances to cause erosion of deciduous enamel, and to determine which chemical factors are most strongly associated with enamel dissolution in deciduous teeth.

## Material and Methods

### Preparation of enamel specimens

From a pool of extracted teeth, we randomly selected 150 caries-free human deciduous molars and 20 (permanent) premolars. The teeth were extracted by dental practitioners in Switzerland. Before the extraction, the patients and their parents were informed about the use of their teeth for research purposes and their oral consent was obtained. Because we are using teeth from a pooled bio-bank, the local ethics committee categorized the samples as “irreversibly anonymised”, and no previous approval was necessary. The crowns of all teeth were separated from the roots, and cut in two halves (into buccal and lingual surfaces). The enamel slabs were embedded in acrylic resin blocks (Paladur^®^, Bad Homburg, Germany) using two planar parallel moulds of 8 mm and 0.2 mm. The latter mould was removed and the blocks were then serially ground (LaboPol-21 rotating polishing machine, Struers, Ballerup, Denmark) with silicon carbide paper discs (grade 18 μm for 30 s, 8 μm grade for 30 s, 5 μm grade for 1 min, 3 μm diamond abrasive paste for 1 min), removing 200 μm of enamel from each specimen. After each polishing step, the resin blocks were rinsed and sonicated for 2 min in tap water and all specimens were then stored in a saturated mineral solution (1.5 mM CaCl_2_, 1.0 mM KH_2_PO_4_, 50 mM NaCl, pH 7.0 [31]) until the time of the experiment. The 300 deciduous enamel samples were randomly distributed into 30 groups (n = 10 for each of the substances tested). The permanent enamel samples were divided into two groups (n = 10).

### Substances tested

In the present study, we tested 30 substances, ranging from drinks, candies, and medicaments frequently used by children and young adolescents (Table 1). For the experiment, all carbonated drinks, candies, and medicaments were pre-treated as follows. The carbonated drinks were degassed by stirring at room temperature (10 min). The candy was dissolved in deionized water (5.2 g candy / 10 ml water), under constant mixing at 45°C; the resulting candy solution was then cooled and used at 30°C for the experiments. The medicaments and concentrated drinks were all prepared with deionized water according to the manufacturer’s instructions. The chewing gum was ground for 5 min (2 g chewing gum in 10 ml of deionized water) using a mortar and pestle, and the resulting solution was used in the experiment. The fruits were squeezed/crushed and the juice was then passed through a sieve (1.0 x 1.0 mm).

**Table 1:** Basic information on the substances tested and their chemical parameters: pH, titratable acidity to pH 5.5 (mmol OH^−^/l to pH 5.5), calcium [Ca], inorganic phosphate [P_i_], and fluoride [F] concentrations, degree of saturation with respect to hydroxyapatite ((p*K*−p*I*)_HAP_), with respect to fluorapatite ((p*K*−p*I*)_FAP_), and with respect to calcium fluoride ((p*K*−p*I*)_CaF2_).

Substance	Brand name/producer	Flavour	Erosion-related ingredients*	pH	mmol OH^−^/l to pH 5.5	[Ca] (mmol/l)	[P_i_] (mmol/l)	[F] (ppm)	(p*K*−p*I*)_HAP_	(p*K*−p*I*)_FAP_	(p*K*−p*I*)_CaF2_
MINERAL WATER											
Mineral water	Valser^®^, Coca-Cola Company	–	–	6.53	–	10.57	< 0.01	0.58	−0.35	3.47	–0.82
SOFT DRINKS											
Coca-Cola^®^	Coca-Cola^®^, Coca-Cola Company	Cola	Phosphoric acid, carbonic acid,	2.55	9.32	0.53	5.39	0.05	−20.59	−14.31	–5.45
Pepsi Cola^®^	Pepsi Cola^®^, PepsiCo	Cola	Phosphoric acid, citric acid, carbonic acid, and flavours	2.51	8.30	0.22	5.38	<0.05	−22.83	−17.09	−7.00
Fanta^®^ Regular	Fanta^®^, Coca−Cola Company	Orange	Orange fruit, citric acid, carbonic acid, and flavours	2.59	36.19	0.56	0.14	<0.05	−24.76	−18.65	−5.64
Sprite^®^	Sprite^®^, Coca−Cola Company	Lemon	Carbonic acid, citric acid, acidity regulator, and flavours	2.57	31.56	0.47	< 0.01	<0.05	−34.71	−28.78	−6.12
Guaraná Antártica^®^	Antártica	Guaraná	Citric acid and carbonic acid	2.62	15.55	0.03	< 0.01	<0.05	−36.96	−31.02	−7.21
Rivella^®^ Red	Rivella	NA	Milk serum, carbonic acid, citric acid, and flavours	3.28	32.88	2.95	2.72	0.07	−12.52	−6.41	−3.61
Ice tea	NA, Coop (supermarket in Switzerland)	NA	Black tea extract, citric and ascorbic acids	2.43	24.36	0.03	0.06	0.88	−33.58	−26.06	−4.45
Ice tea peach	Lipton, Unilever	Peach	Black tea extract and peach juice	2.65	25.15	0.08	0.13	0.55	−28.39	−21.12	−4.01
FRUITS, JUICES AND SMOOTHIES											
Kiwi (fruit)	NA	NA	NA	3.24	159.81	1.06	3.40	<0.05	−14.53	−9.93	−7.12
Orange (fruit)	NA	NA	NA	3.93	71.93	1.50	1.18	<0.05	−10.22	−5.22	−4.77
Orange juice	Hohes C, Eckes AG	Orange	Orange juice	3.63	83.56	2.11	1.58	<0.05	−11.32	−5.89	−4.38
Apple juice	Ramseier Premium, Ramseier Suisse AG	Apple	Apple juice and pear juice	3.24	70.30	1.17	1.62	<0.05	−15.23	−9.44	−4.68
Apple juice for babies	Nestlè	Apple and pear	Apple juice, pear juice, vitamin C	3.59	48.19	2.55	1.96	0.17	−10.98	−4.70	−2.68
Ribena^®^	Lucozade Ribena Suntory	Blackcurrant	Blackcurrant juice concentrate, citric acid, and vitamin C	2.51	27.94	0.36	0.17	0.01	−26.06	−20.42	−6.93
Fruit smoothie	innocent	Kiwi, apple and limes	Apple juice, grape juice, kiwi juice, lime juice, and pineapple juice	3.27	82.44	2.10	0.27	<0.05	−16.13	−10.62	−4.94
YOGHURT											
Forest berries yoghurt	NA, Migros (Supermarket in Switzerland)	Berries	Forest Berries;	4.13	62.86	37.39	10.72	<0.05	−0.55	4.63	−2.86
SOUR CANDIES											
Candy spray	Mega Mouth^®^ Candy Spray, Bazooka Candy Brands International Ltd	NA	Citric acid	2.14	441.75	0.12	0.16	<0.05	−31.67	−26.76	−9.65
Sour candy	Haribo^®^ Pommes, Haribo GmbH & Co.,Germany	Apple	Citric, malic, and tartaric acids	2.46	88.10	0.07	0.12	<0.05	−30.57	−24.64	−7.18
Sour chewing gum	Trident^®^ Senses, Modelez	Mega Mystery	Citric acid, malic acid	2.74	22.57	0.37	0.03	<0.05	−26.56	−21.57	−7.76
SPORTS AND ENERGY DRINKS											
Monster Energy Drink^®^	Monster Energy Drink^®^, Vertrieb Spar GmbH, Austria	NA	Citric, sorbic, carbonic, and benzoic acids, vitamin B, taurine	3.35	62.39	0.07	0.03	<0.05	−25.05	−19.38	−5.82
Red Bull^®^ Energy Drink	Red Bull^®^, Red Bull GmbH, Austria	NA	Sodium citrate, carbonic acid, taurine, vitamin B	3.35	67.76	1.41	< 0.01	0.13	−25.72	−19.38	−3.27
Gatorade^®^	Gatorade^®^, PepsiCo	NA	Citric acid, flavours	2.89	37.38	0.05	2.98	0.05	−23.94	−17.74	−5.97
MEDICAMENTS											
Dafalgan^®^ syrup for children	Bristol−Myers Squibb	NA	NA	5.26	7.91	0.07	< 0.01	<0.05	−15.16	−11.65	−6.37
Mucosolvon^®^ for children	Boehringer Ingelheim	NA	Benzoic acid	3.13	14.43	0.01	0.01	<0.05	−31.47	−26.41	−8.21
Fluimucil^®^ Effervescent	Zambon Schweiz	NA	NA	4.48	14.04	0.01	< 0.01	<0.05	−29.35	−25.55	−8.26
Tossamin^®^ sugar free syrup	Novartis Consumer Health Schweiz	NA	Sorbic acid	4.43	19.46	0.01	1.46	<0.05	−16.42	−12.59	−8.12
Ventolin^®^ syrup	Glaxo Smith Kline	NA	NA	3.19	56.08	0.02	< 0.01	<0.05	−36.98	−32.35	−8.85
Claritine^®^ syrup	MSD Merk Sharp & Dohme AG	NA	Peach aroma	2.98	74.34	0.07	< 0.01	<0.05	−37.13	−32.23	−8.74
Maltofer^®^ syrup	Vifor (International) AG	NA	NA	4.90	5.48	0.12	< 0.01	<0.05	−20.68	−17.47	−7.45

* Erosion-related ingredients are those listed on the packaging of each substance.

NA = not available.

When [P_i_] values were <0.01mmol/l, exact values of 0.0001 mmol/l were used in the (p*K*−p*I*) calculations.

### Chemical analysis of the substances

For the chemical analyses [22], we used 10 g of each solution at 30°C to measure the initial pH and the titratable acidity to pH 5.5 (total amount of base needed to raise the pH of the substance to 5.5). An automatic titrator (Toledo DL 53, Mettler Toledo, Electrode DG 101-SC, Software: LabX pro, Schwerzenbach, Switzerland) established the initial pH of the solutions, which were then individually titrated with 0.5 mol/l NaOH in steps of 0.02 ml [23]. Titratable acidity was calculated as the amount of base (mmol/L of sample) required to raise the pH to 5.5. Calcium (Ca) concentration was measured with the standard atomic absorption method, using an atomic absorption spectrometer with an air/acetylene flame. Lanthanum was added to all the products and standards (final end concentration 0.2%) to suppress interference from inorganic phosphates (P_i_). Total P_i_ concentration was analysed by the ammonium molybdate method of Chen et al. (1956) [32]. Fluoride (F) concentration was determined using an F ion-specific electrode (Orion 960900, Boston, MA, USA). Before F measurement, we added total ionic strength adjustment buffer (TISAB) to all products and standard solutions (1:1 ratio), without previously neutralizing the substances. The concentrations of Ca and P_i_ are expressed in mmol/l and those of F in ppm. The degree of saturation (p*K−*p*I*) with respect to hydroxyapatite (HAP), fluorapatite (FAP), and calcium fluoride (CaF_2_) was calculated from the pH and the concentrations of Ca, P_i_ and F using a computer program [33]. This program assumes a solubility product for HAP of 10^−58.5^ and for FAP of 10^−59.6^ [34, 35]. The concentrations of Ca, P_i_ and F, the pH, and the titratable acidity were measured in duplicate.

### Surface hardness measurement

The present method describes hardness measurements using nanoindentations. Surface hardness (SH) of each enamel specimen was determined with a Vickers diamond under a pressure of 50 mN for 15 s (Fischerscope HM 2000 XYp; Helmut Fischer, Hünenberg, Switzerland). A total of six baseline indentations were made at intervals of 50 μm. Further indentations next to the previous indentations were made following the experimental procedure. Vickers hardness was automatically calculated from the depth of the indentations by the computer program. The load resolution was ≤ 0.04 mN and the indentation depth was 600 nm for sound enamel and < 1000 nm for most softened specimens. The device allowed fully automatic measurements using a programmable x, y stage. The WIN-HCU software calculated SH. The SH value for each enamel slab was determined by calculating the average of six indentations.

### Surface reflection intensity

For the surface reflection intensity (SRI) measurements, we used a recently developed table-top reflection device [36–38]. The device was connected to a computer running a specific software that registers the point of highest reflection intensity, which is expressed as a SRI value. We measured SRI initially (SRI_baseline_) and after the second challenge (SRI_4min_), and from these SRI values, we calculated the relative percentage decrease in reflection intensity (rSRI) using the formula rSRI_i_ = (100×(SRI_4min_−SRI_baseline_)) / SRI_baseline_. In practical terms, more negative rSRI values represent greater decrease in reflection intensity, which, in turn, represent more erosion of the enamel surface.

### Study design

Immediately prior to the experimental procedures, the resin blocks were further polished with 1 μm diamond abrasive for 1 min (LaboPol-6, DP-Mol Polishing, DP-Stick HQ; Struers, Copenhagen, Denmark) to ensure the removal of possible remnants from storage. Initially, the samples were incubated in freshly collected human saliva (20 ml / 6 enamel samples, 3 h, 37°C, under constant shaking). For that, stimulated saliva was collected from one healthy adult donor (stimulated salivary flow rate 2.32 ml/min) by chewing on a piece of paraffin pellets (Fluka; Sigma-Aldrich Chemie GmbH, Munich, Germany) for 30 min. An approval from the institutional review board is not necessary for collecting saliva samples, so the local Ethical Committee (Kantonale Ethikkommission) waived the need for ethical approval. In the eyes of the Ethical Committee, when collecting saliva samples, we are only required to obtain the consent from the saliva expeditor, which can be done verbally. In our study, the saliva donor gave a verbal consent, since written consent was not required. The saliva was collected in an ice-cooled tube at least 1 h after the donor had consumed any food or drink [39, 40]. The samples were then carefully rinsed with tap water (50 s) and with deionized water (10 s), then dried with oil-free air (5 s). All enamel samples had their baseline SH and SRI individually measured (SH_baseline_ and SRI_baseline_), after which they were subjected to two consecutive erosive challenges. Each erosive challenge consisted of individually immersing the specimens into the respective test substance (10 ml / sample) for 2 min at 30°C, under constant agitation (95 rpm). The samples were then taken out of the solution, washed (10 s) and dried (5 s), and a second SH measurement was performed (SH_2min_). Subsequently, the samples were submitted to another erosive challenge (2 min), rinsed, dried, and a final SH and SRI measurement was carried out (SH_4min_ and SRI_4min_). A total of 10 deciduous enamel specimens were tested per substance (5 buccal and 5 lingual surfaces randomly chosen). In addition, the two groups containing the permanent enamel samples were also submitted to the same experimental protocol, and were treated with mineral water (n = 10) or Coca-Cola^®^ (n = 10).

### Statistical analyses

Wilcoxon’s signed rank tests were used to compare the SH and SRI values before and after immersion in the respective drink or solution. Changes in SH (ΔSH) were calculated as follows: for the first 2-min erosive challenge ΔSH_2–0_ = SH_2min_ − SH_baseline_; for the second 2-min erosive challenge ΔSH_4–2_ = SH_4min_ − SH_2min_; and for the total 4-min erosive challenge ΔSH_4–0_ = SH_4min_−SH_baseline_. Associations between the changes in SRI (rSRI, denoted as dependent variable), ΔSH (denoted as the dependent variable) and pH, titratable acidity, and Ca, P_i_ and F concentrations, HAP saturation, FAP saturation, CaF_2_ saturation (independent variables) were investigated using Spearman’s Correlation Coefficients. Since HAP and FAP saturation are not independent of pH, titratable acidity, and Ca, P_i_ and F concentrations, care was taken not to include them in the regression analyses. Multiple linear regression analyses were carried out to verify the association of ΔSH_2−0_ and ΔSH_4−0_ with pH, titratable acidity, Ca, Pi and F concentrations. Association between ΔSH_4−0_ and rSRI were investigated using spearman’s correlation coefficient and linear regression analysis. Furthermore, additional differences between deciduous and permanent enamel were verified using the Mann-Whitney U test. The significance level was set at 0.05 for all analyses.

## Results

**Fig 1:**
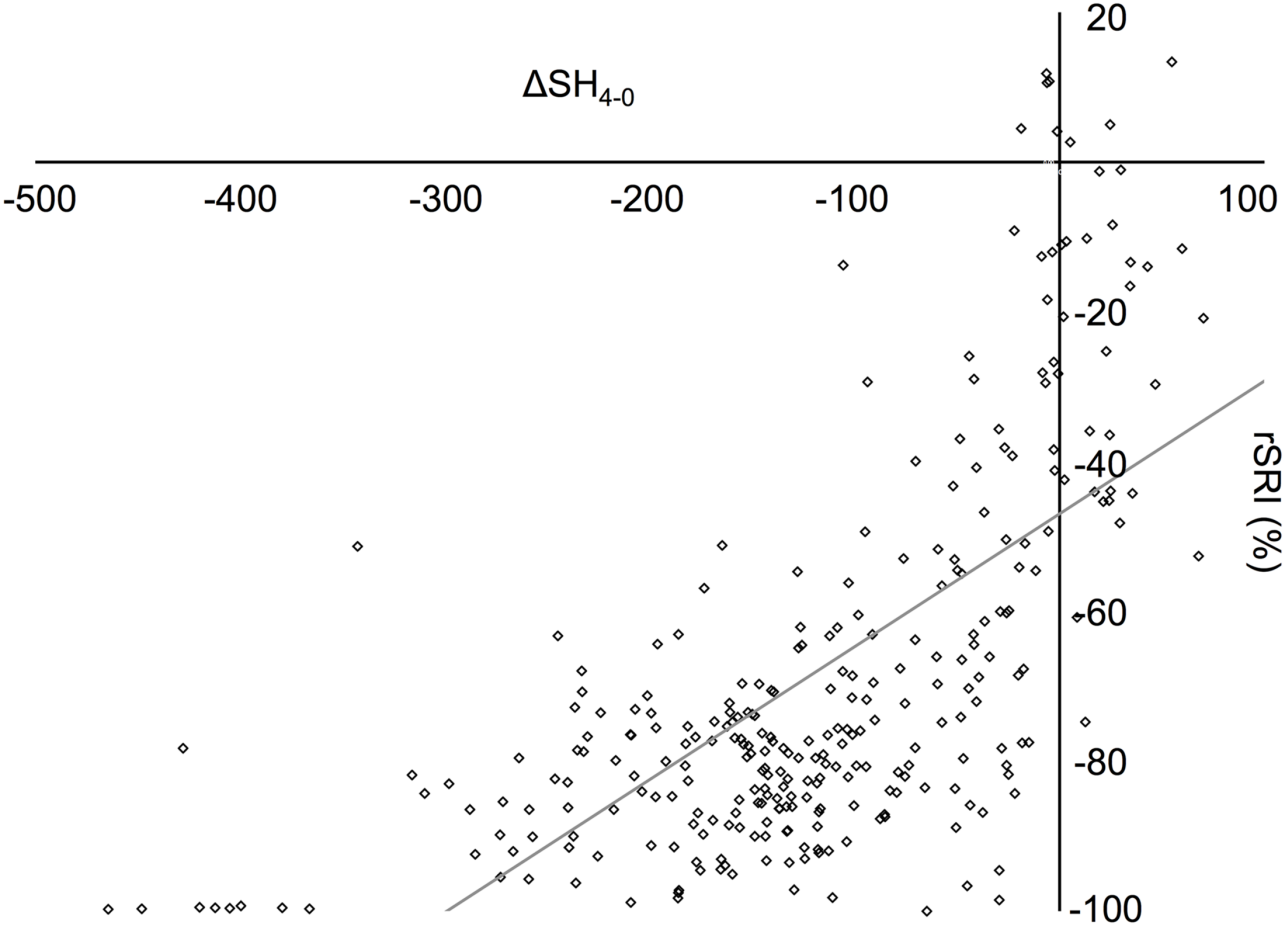
Association between relative surface reflection intensity (rSRI) and change in surface hardness (ΔSH_4−0_). The solid line represents the regression line (Eq 1).

Table 1 presents the 30 substances and their chemical parameters. The SH values at baseline (SH_baseline_), the mean SH loss (ΔSH) after the first (ΔSH_2−0_) and second (ΔSH_4−2_) erosive challenges, as well as the relative surface reflection intensity, are presented in Table 2. Most of the substances caused a significant decrease in SH after the first erosive challenge (p<0.05), with the exception of mineral water (negative control), ice tea peach, apple juice for babies, and some medicaments. Interestingly, during the second erosive challenge, only mineral water, yogurt and some medicaments caused no further loss of SH. After both erosive challenges, all substances caused significant loss in relative surface reflectivity intensity, except for mineral water (Table 2). There was a significant correlation (p < 0.001; ρ = 0.66) between loss in surface hardness (ΔSH_4−0_) and relative percentage decrease in reflection intensity (rSRI; Fig 1), with regression Eq (1) fitting the data:

$$\text{rSRI}=-46.9+0.18\times {\Delta \text{SH}}_{4-0}$$ (1)

**Table 2:** Mean and standard error of the mean (SEM) for surface hardness at baseline (SH_baseline_), the difference in surface hardness between baseline and the first erosive challenge (ΔSH_2−0_), the difference in surface hardness between the first and the second erosive challenges (ΔSH_4−2_), and the relative difference in surface reflection intensity between baseline and the second erosive challenge (rSRI_4−0_).

	SH_baseline_		ΔSH_2−0_			ΔSH_4−2_			rSRI_4−0_		
	Mean	SEM	Mean	SEM	p-value	Mean	SEM	p-value	Mean	SEM	p-value
MINERAL WATER											
Mineral water	509.5	19.6	−5.0	7.7	0.695	−6.1	5.5	0.375	15.6	11.0	0.301
SOFT DRINKS											
Coca−Cola^®^	501.0	12.7	−90.2	11.3	0.002	−79.1	10.3	0.002	−83.0	2.0	0.002
Pepsi−Cola^®^	497.6	10.3	−60.7	7.6	0.002	−86.4	8.8	0.002	−87.7	1.2	0.004
Fanta^®^ Regular	491.2	10.9	−100.6	9.8	0.002	−105.1	14.8	0.002	−85.8	1.5	0.002
Sprite^®^	511.0	13.0	−124.4	4.7	0.002	−134.1	8.2	0.002	−85.3	1.4	0.002
Guaraná Antártica^®^	502.5	14.4	−32.3	8.6	0.014	−58.5	7.8	0.002	−77.1	2.1	0.002
Rivella^®^ Red	491.1	14.2	−44.8	15.0	0.002	−112.9	11.9	0.002	−78.1	2.3	0.002
Ice tea	500.8	10.5	−63.7	5.4	0.002	−84.1	8.4	0.002	−82.8	2.5	0.004
Ice tea Peach	483.6	10.0	−25.5	11.2	0.106	−101.2	9.8	0.002	−82.2	1.6	0.004
FRUITS, JUICES AND SMOOTHIES											
Kiwi (fruit)	498.9	10.3	−60.8	15.9	0.004	−142.3	13.5	0.002	−94.3	1.9	0.002
Orange (fruit)	502.0	11.5	−16.2	5.1	0.014	−43.4	7.3	0.002	−60.7	2.1	0.004
Orange juice	499.4	13.3	−19.2	5.2	0.006	−30.0	5.6	0.002	−72.4	6.1	0.002
Apple juice	480.2	7.6	−37.5	13.6	0.027	−107.4	17.7	0.004	−93.4	1.1	0.002
Apple juice for babies	494.6	9.8	−15.4	7.6	0.065	−48.6	6.0	0.002	−71.0	3.7	0.004
Ribena^®^	506.8	11.3	−50.1	7.0	0.002	−91.4	14.8	0.004	−84.6	2.1	0.002
Fruit smoothie	532.6	15.5	−38.8	10.8	0.006	−77.2	5.3	0.002	−71.6	3.2	0.002
YOGHURT											
Forest berries yoghurt	494.5	6.2	24.7	11.4	0.037	1.6	12.8	0.922	−23.9	7.6	0.006
SOUR CANDIES											
Candy spray	509.9	13.1	−301.7	11.3	0.002	−110.7	12.5	0.002	−97.2	2.4	0.004
Sour candy	525.7	9.0	−74.1	14.3	0.002	−110.7	15.1	0.002	−84.0	2.3	0.002
Sour chewing gum	490.3	13.6	−53.9	7.0	0.002	−81.5	6.7	0.002	−80.7	1.6	0.002
SPORTS AND ENERGY DRINKS											
Monster Energy Drink^®^	509.9	14.9	−51.6	6.7	0.002	−77.1	14.0	0.004	−75.4	2.1	0.002
Red Bull^®^ Energy Drink	515.5	15.3	−52.6	9.3	0.004	−92.2	8.7	0.002	−74.9	2.4	0.002
Gatorade^®^	541.8	18.4	−115.4	20.2	0.002	−89.1	9.2	0.002	−71.7	3.0	0.002
MEDICAMENTS											
Dafalgan syrup	478.9	13.1	17.1	8.4	0.049	18.7	15.6	0.232	−20.6	6.1	0.006
Mucosolvon cough syrup	520.1	13.7	−7.8	9.2	0.625	−41.6	7.5	0.002	−69.9	3.8	0.002
Fluimucil effervescent	496.4	6.8	−11.9	4.7	0.020	−36.1	2.6	0.002	−46.4	4.3	0.006
Tossamin sugar free syrup	510.5	10.3	15.8	14.0	0.492	−13.5	10.6	0.193	−49.5	6.7	0.004
Ventolin syrup	512.9	9.4	−54.2	5.9	0.002	−85.3	6.7	0.002	−74.0	3.7	0.004
Claritine syrup	527.9	15.9	−10.8	5.8	0.106	−13.8	6.4	0.065	−40.8	3.5	0.002
Maltofer syrup	501.7	8.2	9.9	5.7	0.131	−5.4	6.1	0.432	−19.8	4.3	0.002

By far the most erosive substance was candy spray, which caused a loss of SH of more than 300 Vickers Hardness Numbers after the first erosive challenge, and caused the greatest relative change in SRI with a decrease of more than 95% in the SRI of the samples. Kiwi fruit caused the greatest decrease in SH during the second erosive challenge. Regarding the chemical parameters, we see that candy spray had the lowest pH and the highest titratable acidity, whereas kiwi exhibited the second-highest titratable acidity.

Analysing the effect of the different chemical properties of the drinks on dental erosion in deciduous enamel, we see that pH showed a moderate positive correlation with ΔSH and rSRI, whereas all other parameters showed a weak correlation (Table 3). This was also shown by the results of the multivariate linear regression analyses (Table 4), where, despite the weak correlation values observed in Table 3, not only pH, but also titratable acidity, Ca concentration, and, to a lesser extent, Pi concentration all play a role in initial enamel erosion. Table 4 shows that lower pH values and Ca concentration, and higher titratable acidity values are significantly related to more loss of SH during erosion.

**Table 3:** Spearman’s correlation coefficients between the chemical properties of the substances and the difference in surface hardness between baseline and the first erosive challenge (ΔSH_2−0_), the total difference in surface hardness after all erosive challenges (ΔSH_4−0_), and the relative difference in surface reflection intensity between baseline and the second erosive challenge (rSRI_4−0_).

	ΔSH_2−0_	ΔSH_4−0_	rSRI_4−0_
pH	0.635***	0.667***	0.644***
Titratable acidity	−0.197***	−0.275***	−0.256***
[Ca]	−0.018	−0.094	−0.155**
[P_i_]	−0.094	−0.158**	−0.273***
[F]	−0.165**	−0.232***	−0.126*
(p*K*−p*I*)_HAP_ ^±^	0.306***	0.268***	0.153*
(p*K*−p*I*)_FAP_ ^†^	0.289***	0.245***	0.119*
(p*K*−p*I*)_CaF2_ ^‡^	0.029	−0.033	−0.061

[Ca], [P_i_], [F]: calcium, phosphate and fluoride concentrations, respectively;

* significant at p<0.05;

** significant at p<0.005;

*** significant at p<0.001;

^±^ Degree of saturation with respect to hydroxyapatite;

^†^ Degree of saturation with respect to fluorapatite.

^‡^ Degree of saturation with respect to CaF_2_.

**Table 4:** Multiple linear regression analysis of the changes in surface hardness (ΔSH) of all specimens after immersion in all substances.

	Intercept		pH		Titratable acidity		[Ca]		[P_i_]	
ΔSH	*β*	p	*β*	p	*β*	p	*β*	p	*β*	p
ΔSH_2−0_	−135.70	<0.001	34.45	<0.001	−0.46	<0.001	2244.0	<0.001	ns	ns
ΔSH_4−0_	−314.70	<0.001	69.32	<0.001	−0.53	<0.001	3885.0	<0.001	−5457.0	0.023

*β*-estimates and p-values are listed only for variables with a significant impact on ΔSH;

[Ca] and [P_i_]: calcium and phosphate concentrations, respectively;

ns = not significant.

Comparing permanent enamel with deciduous enamel treated with the same substances (Table 5), we observed no significant differences in initial hardness between the two kinds of teeth. However, a significant difference was observed in the change in SH when the samples were immersed in Coca-Cola^®^. After the first erosive challenge (ΔSH_2−0_), deciduous enamel exhibited significantly greater hardness loss (−90.2 ± 11.3 VHN) than permanent enamel (−44.3 ± 12.2 VHN; p = 0.007). However, no differences between the two types of teeth were observed in the total change in SH after both challenges (ΔSH_4−0_), or in the surface reflection intensity.

**Table 5:** Mean ± SEM (standard error of the mean) for surface hardness at baseline (SH_baseline_), difference in surface hardness after the first (ΔSH_2−0_) and both (ΔSH_4−0_) erosive challenges, and the relative change in surface reflectivity (rSRI_4−0_), for deciduous and permanent enamel samples.

Substance		Deciduous	Permanent	p-value
Mineral water (negative control)	SH_Baseline_	509.5±19.6	517.7±11.7	0.280
	ΔSH_2−0_	−5.0±7.7	27.8±13.1	0.089
	ΔSH_4−0_	−11.1±12.0	19.1±12.6	0.089
	rSRI_4−0_	−15.6±11.0	1.6±5.2	0.436
Coca-Cola^®^	SH_Baseline_	501.0±12.7	514.8±13.3	0.579
	ΔSH_2−0_	−90.2±11.3	−44.3±12.2	0.007*
	ΔSH_4−0_	−169.3±11.2	−139.8±10.7	0.075
	rSRI_4−0_	−83.0±2.0	−86.6±1.4	0.143

* Significant difference between deciduous and permanent enamel;

SH_baseline_: surface hardness at baseline;

ΔSH_2−0_: surface hardness decrease between baseline and the first erosive challenge;

ΔSH_4−0_: surface hardness decrease between baseline and the second erosive challenge;

rSRI_4−0_: relative difference in surface reflection intensity between baseline and the second erosive challenge.

## Discussion

Despite the great number of studies on dental erosion, there is still a lack of information regarding the erosive dissolution of deciduous teeth. In the present study, we show the erosive effect of various substances on deciduous enamel. Moreover, we analysed the effect of different chemical factors on the initial erosion process in deciduous teeth. In line with the previous studies, we observed that several soft drinks, fruit juices and smoothies, sour candies, and medicaments can cause significant erosion. This is not surprising given their degree of saturation with respect to HAP and FAP.

Dental enamel is mostly made up of calcium (Ca^2+^), phosphate (PO_4_^3+^), hydroxide (OH^−^), and, to a lesser extent, fluoride (F^−^) ions [41]. In the oral cavity, the teeth are surrounded by saliva, and the enamel crystals are in a constant equilibrium with the saliva. In other words, there is a continuous exchange of Ca^2+^, PO_4_^3+^, OH^−^, and F^−^ between saliva and enamel. When the teeth are exposed to substances that have a low concentration of these ions, there is a tendency for enamel to release more of these ions to the environment in order to attain a new state of equilibrium [41]. Acidic substances with low pH values can exacerbate this process and lead to further demineralization. Therefore, the solubility of enamel is highly dependent on the pH of the surrounding substance, as well as the substance’s Ca^2+^, PO_4_^3+^, and (to a lesser extent) F^−^ concentrations [12, 16, 17, 42–44]. These parameters are, therefore, used to calculate the degree of saturation (p*K*−p*I*) of the substances with respect to hydroxyapatite (_HAP_) and fluorapatite (_FAP_) [33].

The degree of saturation values essentially indicate whether a substance is more or less likely to cause dissolution of enamel. When a substance has (p*K*−p*I*)_HAP_ and (p*K*−p*I*)_FAP_ values below zero, it is said that the substance is undersaturated with respect to HAP and FAP, and this will cause enamel to dissolve until equilibrium is reached. However, if the substance has positive (p*K*−p*I*)_HAP_ and (p*K*−p*I*)_FAP_ values, it is considered supersaturated with respect to HAP and FAP, and will cause ions to deposit on the tooth mineral until a new equilibrium is reached [41]. Interestingly, in the present study, the vast majority of the substances had low pH values (varying from 2.14 to 6.70) and negative (p*K*−p*I*)_HAP_ and (p*K*−p*I*)_FAP_ values, which prompted enamel to demineralize.

Although the (p*K*−p*I*)_HAP_ and (p*K*−p*I*)_FAP_ values are good indicators of whether enamel demineralization occurs, they are calculated based on the ionic composition of HAP and FAP of permanent enamel. Deciduous enamel, however, has a slightly different histological composition, so the (p*K*−p*I*)_HAP_ and (p*K*−p*I*)_FAP_ values presented in Table 1 can only serve as a guide to deciduous enamel dissolution. We therefore carried out the multiple regression analyses to verify which specific variables play a significant role in erosive demineralization of deciduous enamel.

Our results suggest that pH, titratable acidity, Ca^2+^ concentration and, to a lesser extent, P_i_ concentration in the substances can significantly influence erosion in deciduous enamel. Many studies have demonstrated how Ca concentrations in erosive solutions can modulate enamel demineralization [1, 12, 17, 44]. Higher Ca concentration in a given solution will increase its degree of saturation, thus lessening its erosive effect [45]. This is in line with our results, which showed that higher concentrations of Ca in the tested substances prompted significantly less erosive demineralization. P_i_ concentration, on the other hand, was not significant during the first erosive challenge (ΔSH_2−0_), but only became significant after 4 min immersion in the substances (ΔSH_4−0_). Similar results were also observed by Hemingway, Parker (46], who suggested that calcium ions are dissolved from the hydroxyapatite before phosphate ions, thus explaining the relationship between calcium concentration and erosion, and the lack of association between phosphate concentration and erosion. In addition, Lussi, Megert (22] argue that there are four species of P_i_ (H_3_PO_4_, H_2_PO_4_^−^, HPO_4_^2−^ and PO_4_^3−^) that could be present in a solution, but their concentrations are strongly influenced by the pH of the solution. At acidic pH, most P_i_ species are in the form of H_2_PO_4_^−^, and only a minute fraction is in the form of PO_4_^3-^, which is the only species of importance in the ion activity of enamel [22, 46]. Therefore, at low pH, extremely high amounts of P_i_ would be necessary to increase the degree of saturation of a given solution to a level at which it would effectively hinder enamel demineralization [22]. In contrast to what was expected, the multivariate analysis in the present study shows that higher P_i_ concentrations are associated with a greater loss of SH. This is probably because, within the substances we have tested, the highest [P_i_] values were measured in the highly erosive substances, such as Coca-Cola^®^, Pepsi^®^, Rivella^®^ Red, kiwi fruit and Gatorade^®^, and this may be an expression that in some of these substances, like Coca-Cola^®^ and Pepsi^®^, there is a high phosphoric acid content, and, consequently, high Pi concentrations. It is, therefore, possible to conclude that (similarly to permanent enamel) P_i_ concentration does not play a significant role in erosive dissolution of deciduous enamel. Dissolution of deciduous enamel is, thus, strongly influenced by the Ca concentration, pH and titratable acidity of the substance.

Titratable acidity is a measure of the buffering of a solution, and it is directly related to the concentration of the undissociated form of the acid in a given substance [41]. The undissociated form of the acid is of considerable importance because this species has no charge and it is able to diffuse more readily into the near-surface layer of enamel. Once there, this species then dissociates acting as a proton (H^+^) carrier into the enamel mineral, and it maintains the acidic (undersaturated) condition that promotes further dissolution [23, 47]. So, higher titratable acidity values are strong indicators of higher concentrations of the undissociated species of the acid, which, in turn, lead to more enamel erosion.

Besides the effect of specific chemical factors associated with erosion in deciduous enamel, we also compared the effect of two substances (mineral water and Coca-Cola^®^) on both permanent and deciduous teeth. Our results showed no significant differences between the two types of teeth when the specimens were treated with mineral water. Treatment with Coca-Cola^®^, however, caused a significantly greater loss of SH in deciduous enamel than in permanent enamel within the first 2 min (ΔSH_2−0_), but no differences were observed in the total loss of SH after two erosive challenges (ΔSH_4−0_). We, therefore, suggest that the initial erosive process may start differently in the two kinds of teeth, but also the lack of difference after the second erosive challenge could be due to the small sample size in the present study. In any case, conflicting results have been reported from studies on the dissolution pattern of deciduous and permanent enamel [4, 5, 48–52], so these differences should be further investigated.

In the present study, we show that various soft drinks, sour candies, sports drinks and energy drinks, and some fruits and fruit juices are able to cause enamel erosion. Thus, the excessive consumption of such substances can lead to substantial dental erosion, which may compromise patients’ dentition for their entire lifetime [5].

It is important to note that, although the enamel samples were kept in saliva for 3 h to allow the formation of the salivary pellicle, all erosion challenges were made without saliva. More specifically, the sour candy and sour chewing gum were both diluted in water, and the tests on the erosive effect of these substances did not take into account the buffering effect of saliva. In a preliminary experiment carried out in our laboratory, we also dissolved sour chewing gum in human saliva and did the erosive challenge following the methods used in the present study. Dissolving the substance in 10 ml saliva caused no loss in enamel SH after 2 min or 4 min erosive challenge. However, Lagerlof and Dawes [53] showed that the maximum volume of saliva in the mouth before swallowing is 1.19 ml or 0.96 ml for males and females, respectively. So, when the sour chewing gum was dissolved in only 2 ml saliva in the preliminary experiment, we observed that even one drop of the solution was able to considerably decrease enamel SH after 2 min and 4 min challenge, which was probably related to the low pH (3.47) of the solution (unpublished results).

In this experiment, we used two parameters to measure enamel erosion: change in surface hardness (ΔSH) and surface reflection intenstiy (rSRI). Previous studies have shown that SRI is a viable additional method to measure the erosive demineralization of permanent enamel [37, 38, 54], because it highly correlates with Knoop surface microhardness, calcium release, and surface roughness [36]. In the present study, we were able to further demonstrate that SRI is significantly associated with surface hardness measured with Vickers nanoindentations. Moreover, we also show that SRI is a suitable viable option to measure erosive demineralization on deciduous enamel.

In conclusion, we were able to corroborate the erosive potential of a broad range of drinks, foodstuffs and medications commonly consumed/used by children and young adolescents, and we show that erosive dissolution of deciduous enamel is significantly associated with pH, titratable acidity and calcium concentration in the solution. This study is an extensive overview, and it can be used to judge the erosive potential of many dietary substances and medications used by children.
